# Prognosis of Indolent Adult T-Cell Leukemia/Lymphoma

**DOI:** 10.3390/v14040710

**Published:** 2022-03-29

**Authors:** Takuro Kameda, Kotaro Shide, Yuki Tahira, Masaaki Sekine, Seiichi Sato, Junzo Ishizaki, Masanori Takeuchi, Keiichi Akizuki, Ayako Kamiunten, Haruko Shimoda, Takanori Toyama, Kouichi Maeda, Kiyoshi Yamashita, Noriaki Kawano, Hiroshi Kawano, Tomonori Hidaka, Hideki Yamaguchi, Yoko Kubuki, Akira Kitanaka, Hitoshi Matsuoka, Kazuya Shimoda

**Affiliations:** 1 Division of Hematology, Diabetes, and Endocrinology, Department of Internal Medicine, Faculty of Medicine, University of Miyazaki, Miyazaki 889-1692, Japan; takuro_kameda@med.miyazaki-u.ac.jp (T.K.); koutaro_shide@med.miyazaki-u.ac.jp (K.S.); yuuki_tahira@med.miyazaki-u.ac.jp (Y.T.); keiichi_akizuki@med.miyazaki-u.ac.jp (K.A.); ayako_kamiunten@med.miyazaki-u.ac.jp (A.K.); haruko_shimoda@med.miyazaki-u.ac.jp (H.S.); tmnhdk@med.miyazaki-u.ac.jp (T.H.); yamahide@med.miyazaki-u.ac.jp (H.Y.); yoko_kubuki@med.miyazaki-u.ac.jp (Y.K.); 2 Department of Internal Medicine, Koga General Hospital, Miyazaki 880-0041, Japan; masaaki_sekine@med.miyazaki-u.ac.jp (M.S.); hiroshi@kgh.or.jp (H.K.); finesse@kgh.or.jp (H.M.); 3 Department of Internal Medicine, Miyakonojo Medical Center, Miyakonojo 880-8510, Japan; mykgmsato@gmail.com (S.S.); kmtamon@outlook.jp (K.M.); 4 Department of Internal Medicine, Aisenkai Nichinan Hospital, Nichinan 887-0034, Japan; jun31_lord@yahoo.co.jp (J.I.); m-takeuchi@kgh.or.jp (M.T.); 5 Department of Internal Medicine, Miyazaki Prefectural Nobeoka Hospital, Nobeoka 882-0835, Japan; t-toyama@pref-hp.nobeoka.miyazaki.jp; 6 Department of Internal Medicine, Miyazaki Prefectural Miyazaki Hospital, Miyazaki 889-1692, Japan; yamashita@pref-hp.miyazaki.miyazaki.jp (K.Y.); kawanoriaki@yahoo.co.jp (N.K.); 7 Department of Laboratory Medicine, Kawasaki Medical School, Kurashiki 701-0192, Japan; kitanaka@med.kawasaki-m.ac.jp

**Keywords:** adult T-cell leukemia/lymphoma, smoldering-type, chronic-type, iATL-PI

## Abstract

A retrospective chart survey of the clinical features of indolent adult T-cell leukemia/lymphoma (ATL) was conducted in the Miyazaki Prefecture, Japan. This study enrolled 24 smoldering-type ATLs, 10 favorable chronic-type ATLs, and 20 unfavorable chronic-type ATLs diagnosed between 2010 and 2018. Among them, 4, 3, and 10 progressed to acute-type ATLs during their clinical course. The median survival time (MST) in smoldering-type ATL and favorable chronic-type ATL was not reached, and their 4-year overall survival (OS) was 73% and 79%, respectively. Compared with this, the prognosis of unfavorable chronic-type ATL was poor. Its MST was 3.32 years, and the 4-year OS was 46% (*p* = 0.0095). In addition to the three features that determine the unfavorable characteristics of chronic-type ATL, namely, increased lactate dehydrogenase, increased blood urea nitrogen, and decreased albumin, the high-risk category by the indolent ATL-Prognostic Index, which was defined by an increment of soluble interleukin-2 receptor (sIL2-R) of >6000 U/mL, could explain the poor prognosis in indolent ATL patients. The level of sIL-2R might be an indicator of the initiation of therapy for indolent ATL.

## 1. Introduction

Adult T-cell leukemia/lymphoma (ATL) is an aggressive peripheral T-cell lymphoma associated with human T-lymphotropic virus 1 (HTLV-1) infection [1,2,3]. ATL is divided into the following four types according to the Shimoyama classification [4]: acute, lymphoma, chronic, and smoldering. In that report, the 4-year survival rates of the chronic- and smoldering-type ATL were 26.9% and 62.8%, respectively, which were better than those of the acute- and lymphoma-type ATL at 5.0% and 5.7%, respectively. Subsequently, unfavorable prognostic factors, including high lactate dehydrogenase (LDH), high blood urea nitrogen (BUN), and low albumin levels, were identified in chronic-type ATL [5]. As the disease progression of the smoldering- and favorable chronic-type (chronic type without any unfavorable factors) ATL is indolent, they are usually managed with watchful observation until the disease progresses to acute- or lymphoma-type ATL.

Since the initial report, several studies have demonstrated the prognosis of smoldering-and chronic-type ATLs. Katsuya et al. and Imaizumi et al. reported that the 4-year survival rates in smoldering-type ATL were 51.9% and 59.8%, respectively, and that in favorable chronic-type ATL were 60.0% and 62.1%, respectively, while those in unfavorable chronic-type ATL were poor at 29.0% and 26.6%, respectively [6,7]. Takasaki et al. conducted a long-term follow-up of smoldering- and chronic-type ATL, and the 5-, 10-, and 15-year survival rates in their cohort were 47.2%, 25.4%, and 14.1%, respectively [8]. Furthermore, Katsuya et al. proposed a prognostic model for chronic- and smoldering-type ATL that could divide their prognosis into low, intermediate, and high risk by the value of serum soluble interleukin-2 receptor (sIL-2R, U/mL) as ≤1000, >1000 to ≤6000, and >6000, respectively, and their median survival time (MST) of not reached, 5.5 years, and 1.6 years, respectively [6].

In the present study, the prognosis of smoldering- and chronic-type ATL in Miyazaki Prefecture, an HTLV-1 endemic area in southwestern Japan, was investigated, and the usefulness of a previously proposed prognostic model for indolent ATL was evaluated.

## 2. Materials and Methods

### 2.1. Patients

This study retrospectively reviewed the medical records between 2010 and 2018 of patients with smoldering- and chronic-type ATL from seven institutions within Miyazaki Prefecture, an HTLV-1 endemic area in southwestern Japan. The diagnosis of ATL and its subtypes was based on a consensus report and the Shimoyama classification [4].

Detailed clinical data at diagnosis, including the date of diagnosis, age at diagnosis, sex, Eastern Cooperative Oncology Group performance status (PS), presence of skin and lung lesions, complete blood count, abnormal lymphocyte number and percentage in peripheral blood (PB), LDH, BUN, calcium, albumin, and sIL-2R levels were collected. Data on treatment and outcomes were also collected. Overall survival (OS) was calculated from the time of diagnosis to the date of death from any cause or to the last follow-up date. Meanwhile, progression-free survival (PFS) was calculated from the time of diagnosis of smoldering- or chronic-type ATL to the date of progression to acute-type or lymphoma-type ATL, or death by any cause.

### 2.2. Statistical Analysis

Patient characteristics were compared between groups using the χ2 test, Fisher’s exact test, and Mann–Whitney U test. Univariate and multivariate Cox regression analyses of OS were performed for patients for whom sIL-2R was measured at diagnosis. Variables with *p* < 0.1 in the univariate analysis were included in the multivariate analysis. Survival curves were plotted using the Kaplan–Meier method, and the survival rates of each group were compared using the log-rank test. The results were considered statistically significant at *p* < 0.05. All statistical analyses were performed using R v4.0.3.

## 3. Results

### 3.1. Patient Characteristics

Between 2010 and 2018, 54 patients were diagnosed with smoldering- or chronic-type ATL at seven institutions in Miyazaki Prefecture, Japan. Among them, 24 were smoldering-types and 30 were chronic-types (Table 1). The median age at diagnosis of smoldering-type ATL patients was 75.5 years, and that of chronic-type ATL patients was 73 years (*p* = 0.88). Skin lesions were present in 29% of smoldering-type ATL patients and 30% of chronic-type ATL patients. PB leukocyte and lymphocyte numbers were greater in chronic-type ATL patients than in smoldering-type ATL patients (*p* < 0.001 and *p* = 0.006, respectively). Abnormal lymphocytes were detected in PB from smoldering- and chronic-type ATL patients, and their median proportion was 5.25% (interquartile range [IQR] 3.00–8.62, range 0.0–36) and 31.3% (IQR 13.3–53.5, range 0.5–78), respectively. The median number of PB abnormal lymphocytes in chronic-type ATL patients (3.21 × 10^9^/L) was greater than that in smoldering-type ATL patients (0.37 × 10^9^/L). Serum sIL-2R levels were not measured in some of the patients at diagnosis. Among the 13 and 25 cases with smoldering- and chronic-type ATL, respectively, which had data of serum sIL-2R at diagnosis, an elevated level of serum sIL-2R was observed in patients for both ATL types. However, the value in chronic-type ATL (2190 U/mL) was greater than that in smoldering-type ATL (1180 U/mL) (*p* = 0.015).

Among the 30 chronic-type ATL patients, 20 had one or more unfavorable prognostic factors and were categorized as having unfavorable chronic-type ATL; the remaining 10 were categorized as having favorable chronic-type ATL (Table 2). The number of lymphocytes and abnormal lymphocytes did not differ between favorable and unfavorable chronic-type ATL, while the LDH level in unfavorable chronic-type ATL was greater than that in favorable chronic-type ATL (*p* < 0.001) (Table 2). The value of sIL-2R was greater in unfavorable chronic-type ATL (4840 U/mL) than in favorable chronic-type ATL (1595 U/mL), but the difference was not statistically significant (*p* = 0.052).

The median follow-up period for living patients was 5.00 years, 7.32 years, and 4.93 years for smoldering-, favorable chronic-, and unfavorable chronic-type ATL, respectively.

### 3.2. Disease Progression and Therapy

A total of 4 out of 24 smoldering-type ATL patients and 3 out of 10 favorable chronic-type ATL patients progressed to acute-type ATL, with a median time after their original diagnosis of 2.25 years (range 1.53–4.28) and 5.65 years (range 4.94–12.7), respectively. Of these patients, four underwent intensive chemotherapy as part of the VCAP-AMP-VECP regimen, defined as follows: VCAP (vincristine (VCR), cyclophosphamide (CPA), doxorubicin (DXR), and prednisone (PSL))–AMP (DXR, ranimustine, and PSL)–VECP (vindesine, etoposide, carboplatin, and PSL), CHOP (CPA, DXR, VCR, and PSL)-like regimen, or COP (CPA, VCR, PSL) + etoposide with (*n* = 1) or without (*n* = 3) mogamulizumab; one patient received only mogamulizumab therapy, and two patients were treated with oral drugs such as perazolin + etoposide (*n* = 1) and lenalidomide (*n* = 1). The other 27 patients with smoldering- or favorable chronic-type ATL did not progress to acute- or lymphoma-type ATL at the time of final observation. Among them, two smoldering-type ATL patients underwent mogamulizumab therapy alone, and one favorable chronic-type ATL patient underwent a CHOP-like regimen by the physician’s choice since the patient exhibited an increase in sIL-2R levels with the following two or three features: increased LDH value, increased BUN value, and decreased serum albumin level; the patient, however, did not meet the criteria for unfavorable chronic-type ATL and was still categorized as smoldering-type or favorable chronic-type ATL.

Among 20 patients with unfavorable chronic-type ATL, only 1 patient underwent a CHOP-like regimen at diagnosis, and the remaining 19 were carefully monitored without the initiation of chemotherapy. Of them, seven were >80 years old, six patients exhibited PS 3 or 4, two had chronic renal failure, and one had emphysema. Among 19 patients with unfavorable chronic-type ATL who were carefully watched without initiating chemotherapy, 10 progressed to acute-type ATL with a median time of 1.09 years (range 0.02–6.26) after their original diagnosis, and 1 exhibited an increment in serum LDH. Of the 10 patients who progressed to acute-type ATL from unfavorable chronic-type ATL, 5 underwent intensive chemotherapy with a CHOP-like regimen (*n* = 5) with (*n* = 1) or without (*n* = 4) mogamulizumab, 2 patients received only mogamulizumab therapy, and 2 patients were treated with oral drugs such as etoposide (*n* = 1) or perazolin + etoposide (*n* = 1). The remaining one patient did not undergo chemotherapy because of poor PS. One patient who did not receive chemotherapy at the time of diagnosis with unfavorable chronic-type ATL underwent the VCAP-AMP-VECP regimen when the serum LDH level increased during her clinical course.

### 3.3. Prognosis

During the last observation, 6 out of 24 patients with smoldering-type ATLs, 3 out of 10 with favorable chronic-type ATLs, and 12 out of 20 with unfavorable chronic-type ATLs had died. The causes of death for the six patients with smoldering-type ATL were pneumonia (*n* = 5) and ATL (*n* = 1), those for the three patients with favorable chronic-type ATL were ATL (*n* = 2) and multi-organ failure due to cholelithiasis (*n* = 1), and those for the 12 patients with unfavorable chronic-type ATL were ATL (*n* = 8), infection (*n* = 2), other cancer (*n* = 1), and heart failure (*n* = 1).

**Figure 1:**
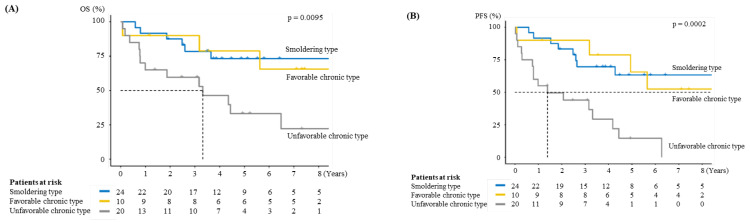
(**A**) Overall survival (OS) and median survival time (MST) by adult T-cell leukemia/lymphoma (ATL) subtype (smoldering, favorable chronic, and unfavorable chronic). (**B**) Progression-free survival (PFS) by ATL subtype.

The MST in smoldering-type ATL and favorable chronic-type ATL was not reached, and their 4-year OS was 73% and 79%, respectively, which was better than that in unfavorable chronic-type ATL, with an MST of 3.32 years and a 4-year OS of 46% (*p* = 0.0095) (Figure 1A).

The 4-year PFS in the smoldering and favorable chronic-type ATL groups was 70% and 79%, respectively. The 4-year PFS rate in the unfavorable chronic-type ATL group was 29%, which was worse than that in the smoldering- and favorable chronic-type ATL (*p* = 0.0002) groups (Figure 1B).

### 3.4. Validation of Indolent ATL-Prognostic Index (iATL-PI)

**Figure 2:**
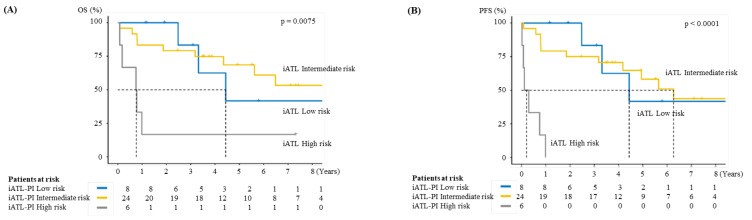
(**A**) Overall survival (OS) by indolent ATL-Prognostic Index (iATL-PI). (**B**) Progression-free survival (PFS) by iATL-PI.

At the time of ATL diagnosis, sIL-2R levels were measured in 38 of the 54 cases. Among them, univariate and multivariate Cox regression analyses of OS confirmed that only the sIL-2R level affected OS (Table 3). The value of sIL-2R is the only factor that is adopted into the iATL-PI, the prognostic model for chronic- and smoldering- type ATL. Six patients were classified as high risk according to iATL-PI, and their MST was 0.77 years, which was worse than that in low- or intermediate-risk patients (*p* = 0.0075) (Figure 2). The PFS of iATL-PI high-risk patients was worse than that of iATL-PI low- or intermediate-risk patients (*p* < 0.0001). All six iATL-PI high-risk patients were chronic-type ATL patients with unfavorable prognostic factors.

## 4. Discussion

This study showed a relatively favorable prognosis for the smoldering- and favorable chronic-type ATL patients, whereas the prognosis of unfavorable chronic-type ATL patients was poor. The reported unfavorable features for the chronic-type ATL were applicable to our cohort, and the iATL-PI, a prognostic model for indolent ATL based on the value of sIL-2R, could efficiently identify patients with poor prognosis among those with indolent ATLs.

In this study, the 4-year OS for smoldering-type ATL was 73% and that for favorable chronic-type ATL was 79%. Compared with the previous reports, in which the 4-year OS for smoldering- or favorable chronic-type ATL was 51.9–62.1% [6,7], this study showed a favorable prognosis for the patients with smoldering- and favorable chronic-type ATL. Moreover, the prognosis of acute- or lymphoma-type ATL in the previous study (MST and 2-year OS were 0.58 years and 23.7%, respectively) was comparable with that from a nationwide survey in Japan [7,9,10,11]. The favorable prognosis for patients with ATL in this study was only observed in the smoldering and favorable chronic-type groups. The median follow-up of surviving patients in this study was 4.93–7.32 years, which was relatively longer than that in previous reports, including the Japanese national survey (4.1–4.3 years) [6,7]. The patient characteristics for smoldering-type ATL between this cohort and the previous report were similar except for the age at diagnosis and proportion of patients with skin lesions [7]. In our cohort, older patients were included more frequently (median age at diagnosis; 75.5 years in this study’s cohort vs. 68 years in the previous report), while patients with skin lesions were included less frequently (29% in this study’s cohort vs. 68% in the previous report [7]). Moreover, the prognosis of patients with smoldering-type ATL and skin lesions was reported to be worse than that of patients without skin lesions [7]. The relatively favorable prognosis in the smoldering-type ATL group in this study might be due to the smaller number of patients with skin lesions.

In this study, the prognosis for unfavorable chronic-type ATL was worse than that for smoldering- or favorable chronic-type ATL. Shimoyama et al. defined poor prognosis features for chronic-type ATL, namely, increased BUN, increased LDH, and decreased albumin values [5]. Accordingly, the Japanese guidelines recommend intensive chemotherapy for patients with chronic-type ATL with one or more of the above unfavorable features [12]. This study was a retrospective chart survey that reflected the clinical practice of patients with ATL. Mainly due to old age, with poor PS, or with organ dysfunction, most of the patients with unfavorable chronic-type ATL in this study did not receive intensive chemotherapy at the initial diagnosis, indicating that the poor prognosis of these patients reflected their natural course and that the above three unfavorable characteristics were useful in predicting the prognosis of patients with chronic-type ATL in clinical practice. Furthermore, the iATL-PI, based on the value of sIL2-R, could efficiently identify patients with indolent ATL with a poor prognosis. The MST for high-risk patients by the iATL-PI was 0.77 years, which was similar to the MST for acute- or lymphoma-type ATL in a previous study [9]. Furthermore, all high-risk patients by the iATL-PI were categorized as having unfavorable chronic ATL. These data demonstrate that high-risk patients with iATL-PI might benefit from early initiation therapy. Since intensive chemotherapy is unsuitable for older patients or patients with poor PS, fewer toxic therapies, such as mogamulizumab, lenalidomide, tucidinostat, or tazemetostat, might be preferable.

Finally, as this study was conducted in a regional cohort, a validation study in a large cohort is needed.

## Figures and Tables

**Table 1:** Clinical characteristics of all patients (*n* = 54).

Variable	Smoldering Type	Chronic Type	*p*
*n*	24	30	
Age (median [IQR])	75.5 (65.5, 80.5)	73.0 [66.0, 81.0)	0.882
Sex, Female/Male, no (%)	8/16 (33.3/66.7)	18/12 (60.0/40.0)	0.094
ECOG PS, no (%)			0.219
0	15 (62.5)	15 (50.0)	
1	6 (25.0)	7 (23.3)	
2	3 (12.5)	2 (6.7)	
3	0 (0.0)	5 (16.7)	
4	0 (0.0)	1 (3.3)	
Skin lesion, absent/present, no (%)	17/7 (70.8/29.2)	21/9 (70.0/30.0)	1
Lung involvement, absent/present, no (%)	23/1 (95.8/4.2)	29/1 (96.7/3.3)	1
WBC count, ×10^9^/L, median [IQR]	6.73 (5.74, 7.48)	12.70 (9.43, 15.19)	<0.001
Neutrophil count, ×10^9^/L, median [IQR]	3.56 (3.01, 4.82)	3.64 (2.72, 4.22)	0.657
Lymphocyte count, ×10^9^/L, median [IQR]	1.63 (1.20, 2.54)	3.22 (1.92, 4.59)	0.006
Abnormal lymphocyte proportion, %, (median [IQR])	5.25 (3.00, 8.62)	31.3 (13.3, 53.5)	<0.001
Abnormal lymphocyte count, ×10^9^/L, median [IQR]	0.37 (0.15, 0.60)	3.21 (2.49, 7.08)	<0.001
Hemoglobin level, g/dL, median [IQR]	12.8 (11.7, 14.3)	12.4 (11.5, 14.0)	0.662
Platelet count, ×10^9^/L, median [IQR]	20.0 (17.0, 24.5)	17.5 (13.0, 22.0)	0.055
Serum TP, g/dL, median [IQR]	6.95 (6.68, 7.53)	6.84 (6.30, 7.27)	0.388
Serum Alb, g/dL, median [IQR]	3.95 (3.70, 4.15)	4.00 (3.70, 4.21)	0.752
BUN, mg/dL, median [IQR]	15.1 (11.0, 17.3)	15.2 (12.6, 19.9)	0.329
Cre, mg/dL, median [IQR]	0.80 (0.64, 1.00)	0.70 (0.60, 0.83)	0.251
Ca, mg/dL, median [IQR]	9.15 (9.00, 9.53)	9.30 (9.03, 9.40)	0.6
T-Bil, mg/dL, median [IQR]	0.60 (0.50, 0.70)	0.70 (0.60, 0.90)	0.056
ALT, IU/L, median [IQR]	17.5 (13.8, 24.3)	20.0 (12.0, 27.8)	0.889
LDH, IU/L, median [IQR]	199 (183,220)	228 (198,291)	0.119
CRP, mg/dL, median [IQR]	0.46 (0.10, 1.53)	0.10 (0.04, 0.40)	0.028
sIL-2R, U/mL, median [IQR]	1180 (930,1730)	2190 (1300, 5040)	0.015
iATL-PI, no (%)			0.121
Low	4 (30.8)	4 (16.0)	
Intermediate	9 (69.2)	15 (60.0)	
High	0 (0.0)	6 (24.0)	

Abbreviations: ECOG PS, Eastern Cooperative Oncology Group performance status; WBC, white blood cells; TP, total protein; Alb, albumin; BUN, blood urea nitrogen; Cre, creatinine; Ca, calcium; T-Bil, total bilirubin; ALT, alanine aminotransferase; LDH, lactate dehydrogenase; CRP, C-reactive protein; sIL-2R, soluble interleukin-2 receptor; iATL-PI, indolent ATL prognostic index; IQR, interquartile range; *p*, *p* value between the smoldering and chronic types.

**Table 2:** Clinical characteristics of chronic-type adult T-cell leukemia/lymphoma (*n* = 30).

Variable	Favorable Chronic Type	Unfavorable Chronic Type	*p*
*n*	10	20	
Age (median [IQR])	70.5 (64.0, 79.0)	74.5 (68.0, 81.0)	0.441
Sex, Female/Male, no (%)	8/2 (80.0/20.0)	10/10 (50.0/50.0)	0.236
ECOG PS, no (%)			0.312
0	7 (70.0)	8 (40.0)	
1	2 (20.0)	5 (25.0)	
2	1 (10.0)	1 (5.0)	
3	0 (0.0)	5 (25.0)	
4	0 (0.0)	1 (5.0)	
Skin lesion, absent/present, no (%)	9/1 (90.0/10.0)	12/8 (60.0/40.0)	0.205
Lung involvement, absent/present, no (%)	10/0 (100/0)	19/1 (95.0/5.0)	1
WBC count, ×10^9^/L, median [IQR]	10.9 (8.75, 17.8)	13.5 (9.88, 14.7)	0.792
Neutrophil count, ×10^9^/L, median [IQR]	3.14 (2.72, 3.99)	3.74 (2.96, 4.37)	0.481
Lymphocyte count, ×10^9^/L, median [IQR]	3.84 (2.30, 5.09)	3.03 (1.39, 4.53)	0.455
Abnormal lymphocyte proportion, %, (median [IQR])	23.0 (8.6, 31.6)	40.0 (19.3, 58.0)	0.179
Abnormal lymphocyte count, ×10^9^/L, median [IQR]	2.72 (1.12, 4.01)	3.83 (2.50, 7.29)	0.356
Hemoglobin level, g/dL, median [IQR]	13.8 (11.8, 14.2)	12.0 (11.6, 13.2)	0.185
Platelet count, ×10^9^/L, median [IQR]	20.0 (17.0, 22.0)	14.5 (12.7, 20.0)	0.333
Serum TP, g/dL, median [IQR]	7.20 (6.93, 7.38)	6.70 (6.20, 7.03)	0.025
Serum Alb, g/dL, median [IQR]	4.20 (4.00, 4.57)	3.80 (3.55, 4.03)	0.012
BUN, mg/dL, median [IQR]	12.7 (10.2, 15.3)	18.5 (13.0, 22.9)	0.014
Cre, mg/dL, median [IQR]	0.60 (0.52, 0.70)	0.80 (0.67, 0.86)	0.039
Ca, mg/dL, median [IQR]	9.25 (9.00, 9.30)	9.30 (9.10, 9.43)	0.58
T-Bil, mg/dL, median [IQR]	0.64 (0.60, 0.78)	0.70 (0.65, 0.92)	0.34
ALT, IU/L, median [IQR]	14.0 (12.0, 26.0)	20.5 (13.5, 28.5)	0.481
LDH, IU/L, median [IQR]	187 (161,202)	260 (231,326)	<0.001
CRP, mg/dL, median [IQR]	0.04 (0.01, 0.06)	0.13 (0.10, 0.57)	0.021
sIL-2R, U/mL, median [IQR]	1595 (1308, 2020)	4840 (1735, 9570)	0.052
iATL-PI, no (%)			0.03
Low	1 (10.0)	3 (20.0)	
Intermediate	9 (90.0)	6 (40.0)	
High	0 (0.0)	6 (40.0)	

Abbreviations: ECOG PS, Eastern Cooperative Oncology Group performance status; WBC, white blood cells; TP, total protein; Alb, albumin; BUN, blood urea nitrogen; Cre, creatinine; Ca, calcium; T-Bil, total bilirubin; ALT, alanine aminotransferase; LDH, lactate dehydrogenase; CRP, C-reactive protein; sIL-2R, soluble interleukin-2 receptor; iATL-PI, indolent ATL prognostic index; IQR, interquartile range; *p*, *p* value between the favorable and unfavorable chronic types.

**Table 3:** Univariate and multivariate Cox regression analyses of overall survival (*n* = 38).

Variable	Univariate		Multivariate	
	HR (95% CI)	*p*	HR (95% CI)	*p*
Age	1.05 (0.99–1.12)	0.110		
Sex, male vs. female	1.02 (0.39–2.66)	0.968		
Subtype, chronic vs. smoldering	2.65 (0.76–9.23)	0.126		
ECOG PS	1.47 (1.02–2.14)	0.041	1.03 (0.63–1.70)	0.898
Skin lesion, present vs. absent	0.59 (0.19–1.82)	0.356		
WBC count, ×10^9^/L ^#^	1.06 (0.97–1.15)	0.203		
Neutrophil count, ×10^9^/L ^#^	1.08 (0.93–1.25)	0.325		
Lymphocyte count, ×10^9^/L ^#^	0.94 (0.78–1.13)	0.509		
Abnormal lymphocyte count, ×10^9^/L ^#^	1.12 (1.00–1.25)	0.050	1.07 (0.90–1.27)	0.434
Hemoglobin level, g/dL	0.89 (0.69–1.15)	0.370		
Platelet count, ×10^9^/L ^#^	1.02 (0.96–1.07)	0.562		
Serum TP, g/dL	0.64 (0.31–1.34)	0.238		
Serum Alb, g/dL	0.45 (0.18–1.14)	0.093	0.42 (0.13–1.44)	0.168
BUN, mg/dL	1.02 (0.95–1.10)	0.586		
Cre, mg/dL	0.91 (0.32–2.61)	0.861		
Ca, mg/dL	0.93 (0.40–2.18)	0.866		
T-Bil, mg/dL	0.72 (0.11–4.52)	0.723		
ALT, IU/L	1.02 (0.99–1.05)	0.277		
LDH, ×10^2^ IU/L ^#^	1.50 (0.93–2.42)	0.101		
sIL2-R, ×10^3^ U/mL ^#^	1.07 (1.03–1.11)	0.001	1.06 (1.01–1.11)	0.010

Abbreviations: ECOG PS, Eastern Cooperative Oncology Group performance status; WBC, white blood cells; TP, total protein; Alb, albumin; BUN, blood urea nitrogen; Cre, creatinine; Ca, calcium; T-Bil, total bilirubin; ALT, alanine aminotransferase; LDH, lactate dehydrogenase; sIL-2R, soluble interleukin-2 receptor; *p*, *p* value; HR, hazard ratio; CI, confidence interval. ^#^, Hazard ratios are given for a 1 × 10^9^/L-increase in WBC, neutrophil, lymphocyte, abnormal lymphocyte, and platelet count, a 100-unit increase in LDH, and a 1000-unit increase in sIL-2R.

## Data Availability

The data supporting the findings of this study are available upon request from the corresponding author. The data were not publicly available because of privacy or ethical restrictions.
